# Hydrolysis of a second Asp-Pro site at the N-terminus of NOTCH3 in inherited vascular dementia

**DOI:** 10.1038/s41598-021-96679-9

**Published:** 2021-08-26

**Authors:** Xiaojie Zhang, Soo Jung Lee, Michael M. Wang

**Affiliations:** 1 Department of Neurology, University of Michigan, 7725 Medical Science Building II Box 5622, 1137 Catherine St., Ann Arbor, MI 48109-5622 USA; 2 Department of Molecular and Integrative Physiology, University of Michigan, Ann Arbor, MI 48109 USA; 3 Neurology Service, Department of Veterans Affairs, VA Ann Arbor Healthcare System, Ann Arbor, MI 48105 USA

**Keywords:** Stroke, Dementia, Stroke, Dementia, Neurodegeneration

## Abstract

Cerebrovascular pathology at the biochemical level has been informed by the study of cerebral autosomal dominant arteriopathy with subcortical infarcts and leukoencephalopathy (CADASIL), a vascular disorder caused by *NOTCH3* mutations. Previous work in CADASIL described N-terminal proteolysis of NOTCH3 generated by specific non-enzymatic cleavage of the first Asp-Pro sequence of the protein. Here, we investigated whether the second Asp-Pro peptide bond (residues 121–122) of NOTCH3 is cleaved in CADASIL. Monospecific antibodies were generated that recognize the neo-epitope predicted to be generated by cleavage after Asp121. These antibodies were used to localize cleavage events at Asp121 in post-mortem CADASIL and control brain tissue and to investigate factors that regulate cleavage at Asp121. We report that cleavage at Asp121 occurs at a high level in the arterial media of CADASIL cerebral arteries. Leptomeningeal arteries demonstrated substantially more cleavage product than penetrating arteries in the white matter, and control vessels harbored only a small amount of cleaved NOTCH3. Proteolysis at Asp121 occurred in purified preparations of NOTCH3 ectodomain, was increased by acidic pH and reductive conditions, and required native protein conformation for cleavage. Increasing the concentration of NOTCH3 EGF-like domain protein elevated the level of proteolysis. On the other hand, several polyanionic chemicals potently blocked cleavage at Asp121. These studies demonstrate that the NOTCH3 protein in CADASIL is cleaved in multiple locations at labile Asp-Pro peptide bonds. As such, chronic brain vascular disease, like other neurodegenerative conditions, features proteolysis of pathological proteins at multiple sites which may generate small pathological peptides.

## Introduction

Although intrinsic vascular disease of the brain is a major cause of stroke and an amplifier of age-related neurodegeneration, an understanding of the underpinnings of brain artery pathology at the biochemical level is still evolving. CADASIL, the leading inherited cause of small vessel disease, provides an attractive model to understand the pathogenesis of vascular pathology because it affects brain vessels with reduced influence of confounding age-related factors such as atherosclerosis and amyloidosis^1,2^. CADASIL results from cysteine-altering mutations in *NOTCH3*^3,4^ which has placed a spotlight on NOTCH3 protein alterations. Prior work has shown that the NOTCH3 protein accumulates in CADASIL vessels^5^; moreover, antibodies developed to detect conformational alterations of NOTCH3 preferentially bind to degenerative CADASIL arteries^6^. More recently, cleavage of NOTCH3 between the first and second EGF-like repeats has been identified in degenerating arteries of CADASIL^7^.

The cleavage of NOTCH3 at its N-terminus has been characterized in vitro^7^. The protein is cut at an Asp-Pro bond and is abrogated in recombinant proteins in which the proline residue is mutated. In addition, in recombinant protein, mutations of multiple cysteine residues of NOTCH3 or treatment of protein with reductants enhance cleavage. Because cleavage occurs in purified preparations of protein, it is likely that this site specific proteolytic event occurs independent of proteases.

In other neurodegenerative conditions, pathogenic proteins undergo proteolysis in multiple sites^8–13^. In addition, several other proteins have been described that are cleaved at Asp-Pro bonds^14–19^, and it is well-known that this dipeptide sequence is labile, particularly under acidic conditions^14–17,19^. With this consideration, we investigated in this study whether a second Asp-Pro site in the N-terminus of NOTCH3 undergoes cleavage in CADASIL.

## Results

### Development of probes to detect a second N-terminal cleavage site in NOTCH3

**Figure 1:**
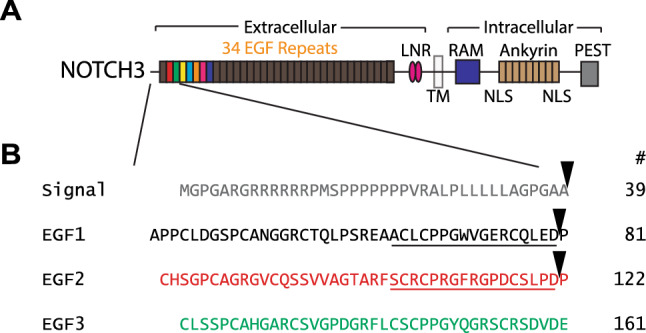
Domain structure and primary amino acid sequence of the N-terminus of NOTCH3. (**A**) Schematic representation of NOTCH3 protein showing N-terminal series of 34 EGF-like domains that compose the bulk of the ectodomain. The vast majority of mutations that cause CADASIL are in the N-terminal repeats that are shown in multiple colors. (**B**) The primary sequence of the N-terminus of NOTCH3 that includes the signal peptide and first three EGF-like repeats. The junction between the first (black) and second (red) and between the second and third (green) EGF-like domains contain Asp-Pro sequences (residues 80–81 and 121–122). Potential cleavage sites are designated by inverted triangles; the right column displays the amino acid position of the terminal residue of each line. The sequence terminating with Asp80 has been shown before to be cleaved^7^. The sequence underlined in black (referred to as the E1 peptide later) was previously used to generate UMI-D and UMI-F antibodies. The neo-epitope generated by the predicted cleavage after Asp121 at the end of the second EGF-like domain is underlined in red (called the E2 peptide later). A peptide corresponding to this neo-epitope was used to generate antibodies 83G, 120B, and 145H.

We hypothesized that in addition to the first Asp-Pro sequence of NOTCH3, additional Asp-Pro sequences of the protein could be vulnerable to proteolysis. The current work focuses on the Asp-Pro sequence that separates EGF-like domains 2 and 3 in NOTCH3 (Fig. 1). Cleavage at this novel site is predicted to generate a novel C-terminal epitope that terminates in an aspartic acid residue (human residue Asp121; Fig. 1). In order to detect cleavage at this novel site, monoclonal antibodies specific for the neo-epitope created by this specific cleavage were generated.

**Figure 2:**
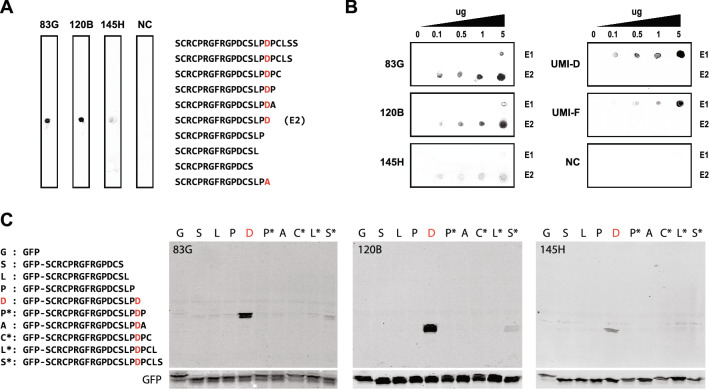
Monoclonal antibodies to a neo-epitope produced by cleavage between the second and third EGF-like domains of NOTCH3. Monospecific reagents were selected based on binding to the neo-epitope ending in Asp121. (**A**) The specificity of three antibodies used in this study were characterized by probing dot blots of peptides with the sequences shown. NC was a negative control sample containing irrelevant rabbit antibody. The terminal residue of the neo-epitope revealed by cleavage after Asp121 is shown in red. The peptide used for immunization is denoted E2. The peptide at the bottom includes a mutation of Asp121 to alanine highlighted in red. (**B**) Monoclonal antibody reactivity to the neo-epitope from Asp-Pro cleavage at the end of EGF-like domain 2 was compared to reactivity against the neo-epitope from Asp-Pro cleavage at the end of EGF-like domain 1 that was previously shown to be a cleavage site (see Fig. 1). Peptides at denoted doses corresponding to neo-epitope ending in Asp80 (E1; top row of panels) and neo-epitope ending in Asp121 (E2; bottom row left three panels) were probed with antibodies shown. UMI-D and UMI-F antibodies were previously described as probes against the neo-epitope ending in Asp80^7^. (**C**) Western blot analysis of protein lystates from cells transfected with GFP fusions designed to generate proteins with variable C-terminal residues shown. The C-terminal Asp121 residue predicted to be generated by Asp-Pro cleavage is shown in red. Asterisks denote constructs that include wildtype amino acid extensions beyond the neo-epitope generated by Asp-Pro cleavage. The sequences of the C-terminal extension of GFP are shown in the left panel. The blots were probed with antibodies denoted in the upper left corner of the large blot. The small blot below shows GFP signal from the same membrane.

A series of thirteen monoclonal antibodies that reacted with the immunizing peptide by ELISA were analyzed by assessing (1) reactivity to peptides corresponding to variants of the C-terminal neo-epitope by dot blotting; (2) binding to the neo-epitope from Asp-Pro cleavage after domain 2 but not after domain 1; and (3) reactivity to fusions of GFP linked at its C-terminus to fragments of EGF-like domain 2 of NOTCH3 by Western blotting. All except one of these monoclonal antibodies strongly preferred the C-terminal neo-epitope sequence ending in Asp121. The monoclonal antibodies 83G, 120B, and 145H were used in further studies to characterize NOTCH3 cleavage. Dot blotting disclosed that these antibodies recognized peptides ending in Asp121 and that deletion or alanine substitution of Asp121 or extension of the sequence beyond Asp121 eliminated detectable binding (Fig. 2A). These antibodies were used on dot blots that compared specificity for the neo-epitopes generated by cleavage at Asp80 and Asp121 (which are generated by cleavage after EGF-like domains 1 and 2, respectively; see Fig. 1 underlined sequences). All three antibodies recognized neo-epitope ending in Asp121 (E2) but not the neo-epitope ending in Asp80 (E1) of NOTCH3 (Fig. 2B) except at the highest concentration of peptide. Western blotting of GFP fusions ending in EGF-like domain 2 sequences revealed that all monoclonal antibodies specifically recognized epitopes that precisely ended in Asp121 and did not recognize proteins with deletion of Asp121 or extensions beyond Asp121 (Fig. 2C). Antibodies detected protein in 293 cells that were transfected with GFP fusions encoding peptides ending in Asp121 but not with fusions that ended in Pro120 or Pro122 (Supplementary Fig. 1).

### Cleavage of NOTCH3 at Asp121 in CADASIL

**Figure 3:**
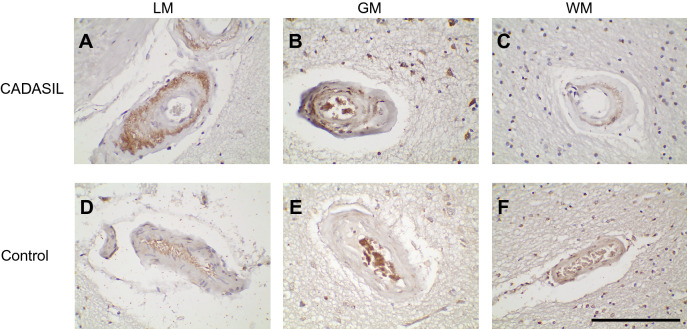
Localization of a second Asp-Pro cleavage site in NOTCH3 to cerebral arterial media in CADASIL. Immunohistochemistry using monoclonal antibody 145H at 0.6 µg/ml against the neo-epitope ending in Asp121 was performed on paraffin sections from CADASIL (**A**–**C**) and control (**D**–**F**) brain. Images of leptomeningeal (LM), gray matter (GM), and white matter (WM) arteries are shown. Additional CADASIL and control samples that were examined are shown in Supplementary Figs. 3 and 4. The scale bar that applies to all images marks 100 microns.

To determine whether cleavage of NOTCH3 at Asp121 of the human protein occurs in CADASIL brain, we performed immunohistochemistry using antibodies against the neo-epitope terminating in Asp121. Each of the three antibodies (83G, 120B, and 145H) reacted strongly with the arterial media of vessels in CADASIL brain (Supplementary Fig. 2). For two of the antibodies, there was occasional signal observed in neurons of both CADASIL and control samples (Supplementary Fig. 2, 83G and 120B with 10× magnification on the far right side). Therefore, we used 145H for all subsequent IHC analysis. Analysis of a set of 20 CADASIL samples and 13 controls were performed with 145H (see Supplementary Table 1 for sample characteristics). Staining for the neo-epitope terminating in Asp121 by 145H was observed in all CADASIL samples but one and was strongly localized to arteries (Supplementary Figs. 3 and 4). Use of 145H demonstrated medial staining that was notably stronger in CADASIL (Fig. 3A–C) compared to control arteries (Fig. 3D–F).

Leptomeninges (LM; Fig. 3A) had the highest levels of protein deposition in CADASIL, and within leptomeningeal vessels, there was also predominantly medial staining. The overall level of staining in CADASIL vs control in LM was 1.575 vs 0.462 (Supplementary Table 1; scoring value 0: none; 1: staining in less than 50% vessels; 2: staining in over 50% vessels; p < 0.0001). Accumulation of staining in the media was determined using ImageJ; for CADASIL and control vessels shown in Supplementary Fig. 3, the average ratio of medial to adventitial staining were 0.22 ± 0.09 and 0.10 ± 0.05 (p < 0.001).

Gray matter (GM) arteries were also stained with 145H in CADASIL (Fig. 3B). Penetrating white matter (WM) arteries (Fig. 3C) were stained in 15 out of 20 CADASIL patients, with the remainder of patients showing no white matter vascular staining using standardized staining conditions described. These studies are consistent with the possibility that NOTCH3 is cleaved at Asp121 in arteries with principle deposition in LM arterial media of CADASIL patients.

**Figure 4:**
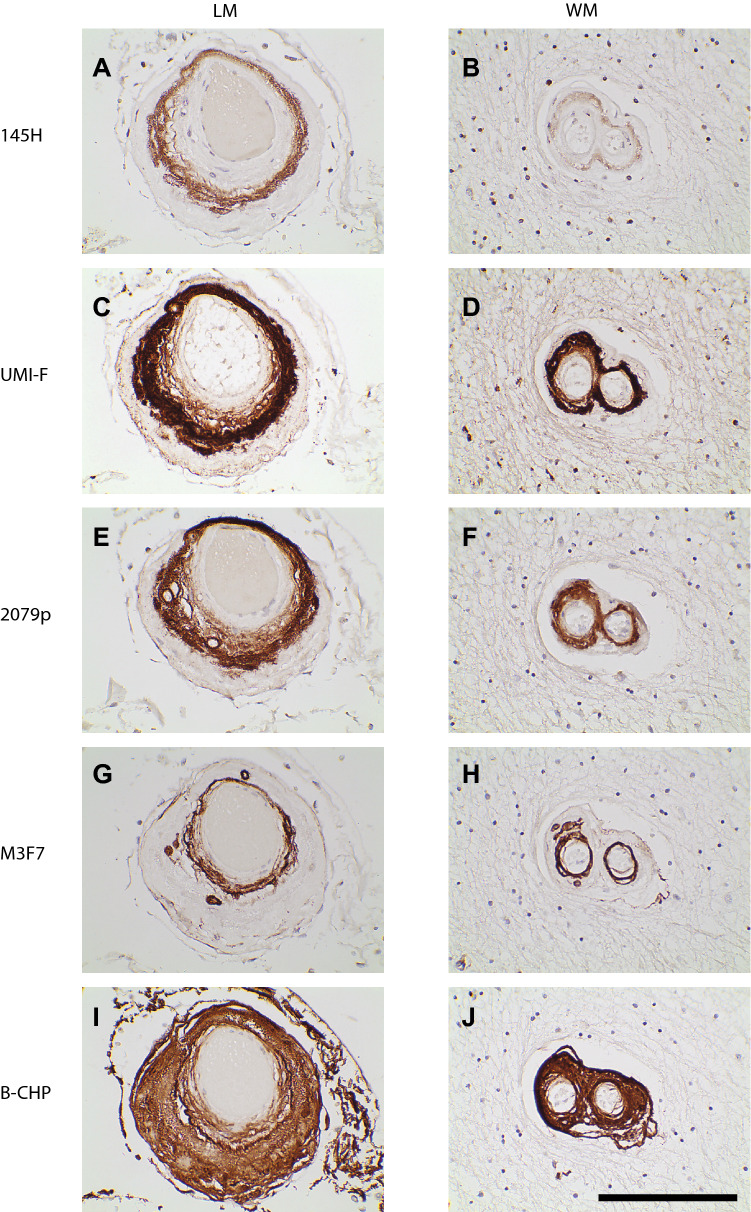
Localization of Asp121 cleavage with other NOTCH3 transformations and collagen. Serial sections from CADASIL brain show LM and WM brain regions stained with antibodies denoted. Consecutive 5 micron slices from the same block were stained with probes shown and representative images were captured. The LM and WM images are from the same slide. 145H recognizes the Asp121 neo-epitope (0.6 µg/ml), 2079 stains reduced NOTCH3^6^ (1:500 dilution), UMI-F recognizes the Asp80 neo-epitope^7^ (undiluted supernatant from transfected 293 cells), M3F7 recognizes type IV collagen (1:50 dilution), and B-CHP recognizes denatured collagen^21^ (1:50 dilution). Scale bar represents 100 microns for all images.

To determine the spatial relationship between NOTCH3 proteolytic products ending in Asp121 and Asp80 in CADASIL arteries, we stained serial sections with 145H and UMI-F^7^ (UMI-F is the monoclonal antibody specific for the neo-epitope revealed by cleavage of NOTCH3 after Asp80; Fig. 4A–D). This revealed that both proteolytic products localized to the same region of both LM and WM arteries, concentrating in the media with much lower levels in the intima. Comparison of LM and WM images taken from the same slide also demonstrates that 145H reactivity was much higher in LM compared to WM; however, UMI-F reactivity was similar between the same LM and WM vessels.

Reduced NOTCH3 protein (revealed by staining with 2079p^6^) was deposited in the same areas of LM and WM vessels as Asp121 cleavage (Fig. 4E–F). Like Asp80 cleavage products, levels of reduced NOTCH3 were similar in LM and WM vessels.

We also assessed for potential co-localization between Asp121 cleavage and type IV collagen in CADASIL vessel in adjacent sections. In the media, type IV collagen was deposited in localized sub-regions of the media, with the heaviest localization in ballooned smooth muscle cells. In contrast, the entire circumference of the media featured expression of Asp121 cleavage (Fig. 4A,B,G,H, and Supplementary Fig. 5). In the intima, we noted significant deposition of type IV collagen as noted before^20^. In contrast, there was heterogeneous intimal staining for Asp121 cleavage that was significantly less intense than medial staining (see 145H staining in Supplementary Fig. 2). Total collagen, defined by binding to the B-CHP peptide probe^21^, was present in the entire artery, including the adventitia, media, and intima, showing that cleavage at Asp121 occurred only part of the area where collagen accumulated (Fig. 4A,B,I,J, and Supplementary Fig. 5).

### Characterization of cleavage at Asp121 of NOTCH3

**Figure 5:**
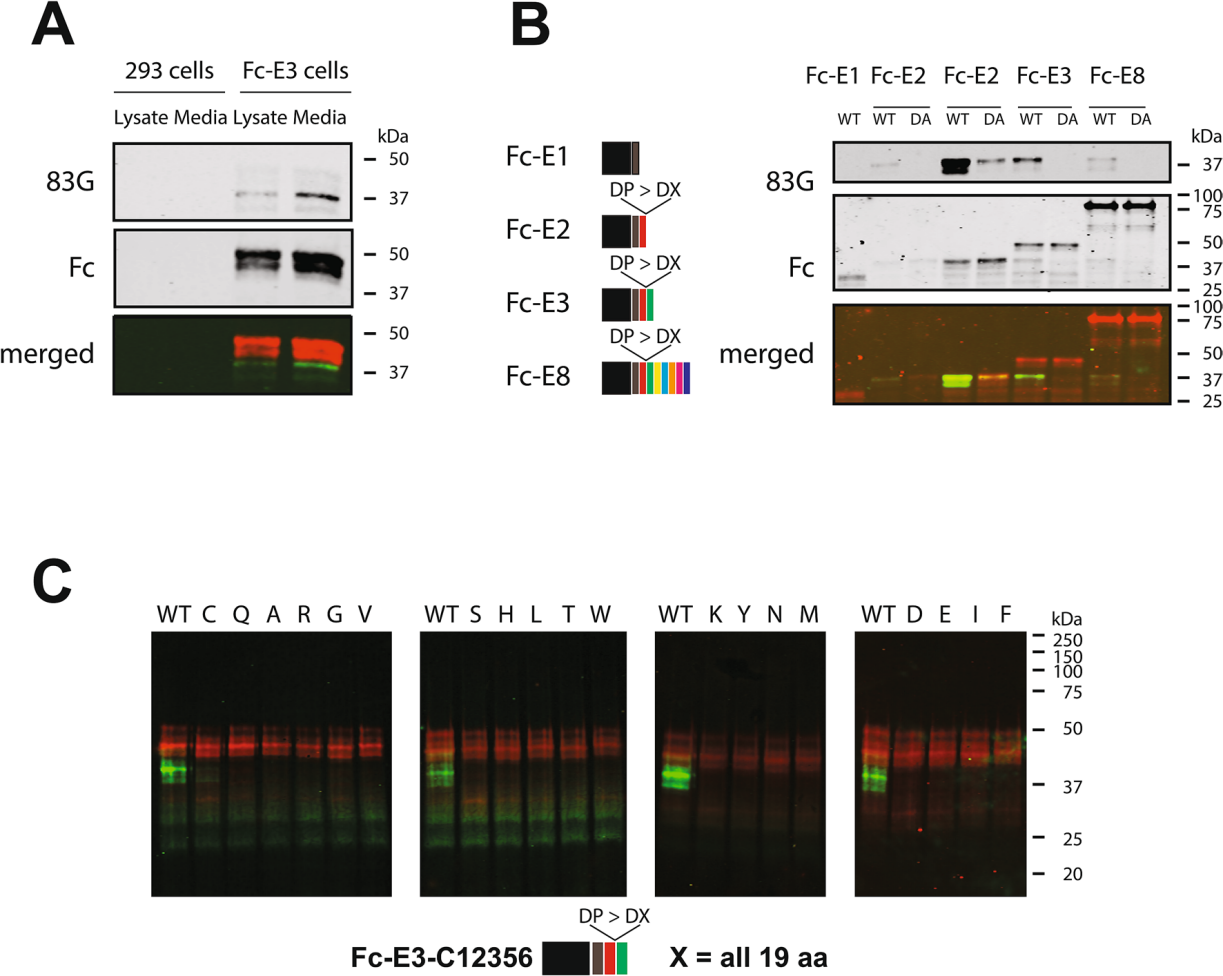
Cleavage at Asp-Pro junctions of recombinant NOTCH3 produced in cells. Three NOTCH3 N-terminal EGF-repeats were cloned C-terminal to Fc to enable purification of by protein A agarose beads; this protein is denoted Fc-E3. (**A**) A 293 cell line expressing Fc-E3 construct encoding Fc fused to the first three EGF-like repeats of NOTCH3 was analyzed for cleavage between the second and third repeats using 83G. We compared this to an identical analysis of control 293 cell proteins. Lysates and conditioned media were purified using protein A beads and were analyzed by western blotting for both cleaved protein (by 83G analysis, green bands at 38 kDa) and intact protein (by Fc analysis, red bands at 42 kDa). (**B**) Recombinant proteins corresponding to constructs depicted on the left were collected from transfected cell media and purified for Western analysis with 83G to detect the neo-epitope ending in Asp121. In the constructs containing more than one EGF-like repeat, the proline following Asp121 was mutated to Ala to determine the requirement for Pro122 in cleavage at the second Asp-Pro sequence (DA). Fc fusions with NOTCH3 fragments that included 1, 2, 3, or 8 EGF-like domains were analyzed by Western blotting with 83G. The blots were also probed for mouse Fc to assess total protein analyzed. For Fc-E2, two paired WT (wildtype) and DA samples are shown; the left one contains one tenth the amount of Fc reactive protein relative to the right in order to allow comparison of the efficiency of cleavage. (**C**) A series of constructs containing Fc and three EGF-like domains (Fc-E3-C12356) were generated (see “Methods”) contained systematic alterations at residue 122 (WT is Pro122). Pro122 was mutated to all 19 other amino acids shown above lanes to determine the requirement for Pro122 in cleavage. The proteins were captured by protein A beads and treated with 50 mM Tris pH 3.0. Constructs contained multiple cysteine mutations to enhance the ability to detect small amounts of protein cleavage.

A 293 cell line that stably expresses a construct encoding Fc fused to the first three EGF-like repeats was generated. The conditioned media of the cells contained an Fc protein band at the expected molecular weight that was not found in untransfected cells (Fig. 5A). When the protein was analyzed by 83G immunoblotting, a faster migrating band was detected that ran at the expected molecular weight of Fc plus two EGF-like repeats. This suggested that the protein is cleaved when produced in cultured cells. The fragmented protein was found in both the cell lysate and the media indicating that cleavage was not exclusive to the extracellular space.

To determine if the NOTCH3 sequence could fragment in other contexts, we generated protein by transient transfection of additional constructs that included the second NOTCH3 Asp-Pro site. Fragments of NOTCH3 that include EGF-like domains 1, 1 through 2, 1 through 3 or 1 through 8 were cloned as a fusion to Fc and expressed in mammalian cells (proteins Fc-E1, E2, E3 and Fc-E8; schematic on the left of Fig. 5B). Western blot analysis of purified proteins using anti-Fc antibodies revealed migration at the expected molecular weight on SDS-PAGE (Fig. 5B). When blots were probed with 83G, the full length protein was not recognized. Instead, fragments corresponding to the first two EGF-like repeats was identified in both Fc-E3 and Fc-E8 (but not Fc, Fc-E1) protein preparations; these fragments migrated at the same size and co-migrated with recombinant Fc-E2 protein composed of Fc plus the first two EGF-like domains ending in Pro121 (WT lanes; Fig. 5B). Additional proteins with mutations at Pro122 were generated for Fc-E2, Fc-E3, and Fc-E8 (DA lanes; Fig. 5B). These proteins contained dramatically reduced amounts of neo-epitope for Fc-E2 and no detectable neo-epitope for Fc-E3 and Fc-E8 (see Supplementary Fig. 6 for explanation). This indicated that NOTCH3 fragmentation at Asp121 occurs spontaneously in cells and is largely dependent on the Asp-Pro sequence. Antibody 83G was used for Western blots because it generated the strongest signals while maintaining specificity for proteins that ended with Asp121. However, all three monoclonal antibodies (83G, 120B, and 145H) detected an Fc-E2 sized band in Fc-E2, Fc-E3 and Fc-E8 samples (Supplementary Fig. 7).

The importance of Pro122 for cleavage was determined by site-directed mutagenesis of Fc-E3. Constructs were created with three EGF-like repeats and in which Pro122 was altered to all other 19 natural amino acids. To increase the sensitivity of the assay, proteins were treated with pH 3.0 buffer (50 mM Tris) and additional mutations of Fc-E3 were created in cysteines to enhance cleavage as described^7^. After expression in 293 cells, Fc tagged NOTCH3 fragments were purified by protein A agarose and acid elution and then analyzed by Western blotting using 120B to detect cleavage after Asp121. Of the 20 amino acids encoded at residue 122, only proline demonstrated cleavage (Fig. 5C).

**Figure 6:**
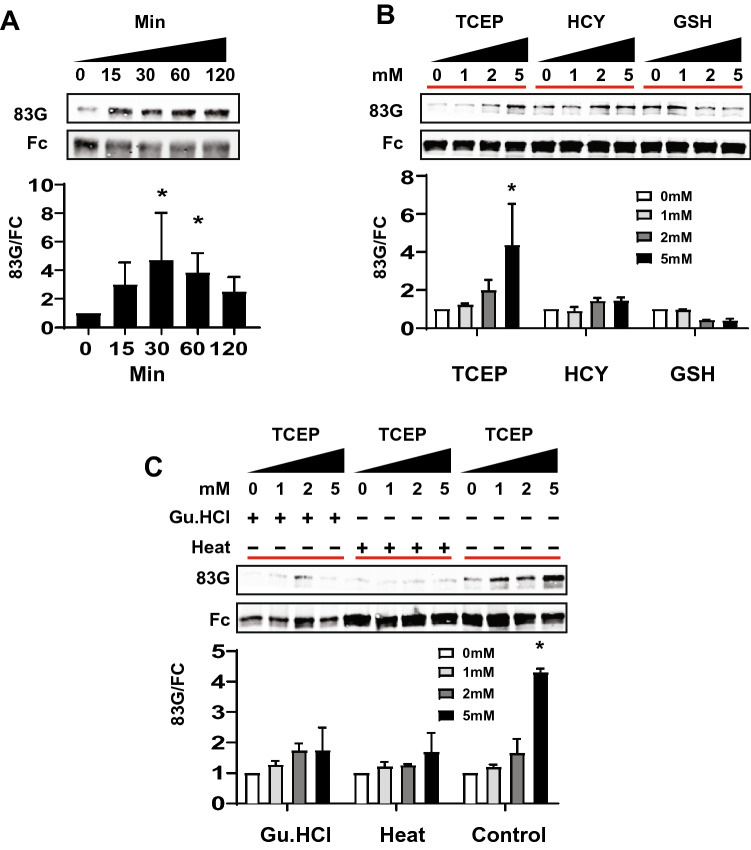
Effect of redox and denaturation on purified NOTCH3 ectodomain cleavage. (**A**) Time course of generation of neo-epitope from purified Fc-E3 protein. Protein was incubated at 37 °C for specific periods of time in TCEP (2 mM) and the analyzed by immunoblotting using probes denoted. 83G was used to quantify neo-epitope released from Asp121 cleavage; Fc was determined on the same blot to normalize protein in each lane. Quantification from five experiments is shown below. (**B**) Fc-E3 protein containing Fc linked to the first three EGF-like domains of NOTCH3 were exposed to increasing concentrations of reductants shown (homocysteine [HCY] and reduced glutathione [GSH]). Western analysis was performed on proteins with 83G to reveal the amount of cut protein and with Fc to normalize for total loaded protein. The ratio of cut to total input protein is displayed below, calculated from three experiments. (**C**) Protein denaturation by chaotropic agents (guanidine HCl [GnHCl] at 1 M) or heat (65 °C for 60 min) was performed prior to the addition of TCEP at specified concentrations to determine whether native structures are necessary for proteolysis at Asp121 (detected by 83G). Total protein was normalized by probing for Fc and the ratio of 83G/Fc determined in three experiments.

We tested the effect of redox conditions (which favor cleavage at Asp80^7^) on Asp121 cleavage (Fig. 6). Strong reducing agents including TCEP markedly increased cleavage at Asp121 of purified Fc-E3 protein within 15 min of treatment (see 83G western blot; Fig. 6A); there was significantly increased cleavage after 30 and 60 min, although the majority of the protein remained uncleaved (see Fc; Fig. 6A). In contrast, mild reducing agents including homocysteine (HCY) and reduced glutathione (GSH) did not alter the magnitude of cleavage (Fig. 6B). To test whether native protein conformation is necessary for reduction mediated cleavage, we challenged protein with sequential denaturation followed by reduction with TCEP. The cleavage of Fc-E3 induced by TCEP was blocked by pre-treatment with heat and pre-treatment with chemical denaturants that are predicted to disrupt the structure of the protein (Fig. 6C).

**Figure 7:**
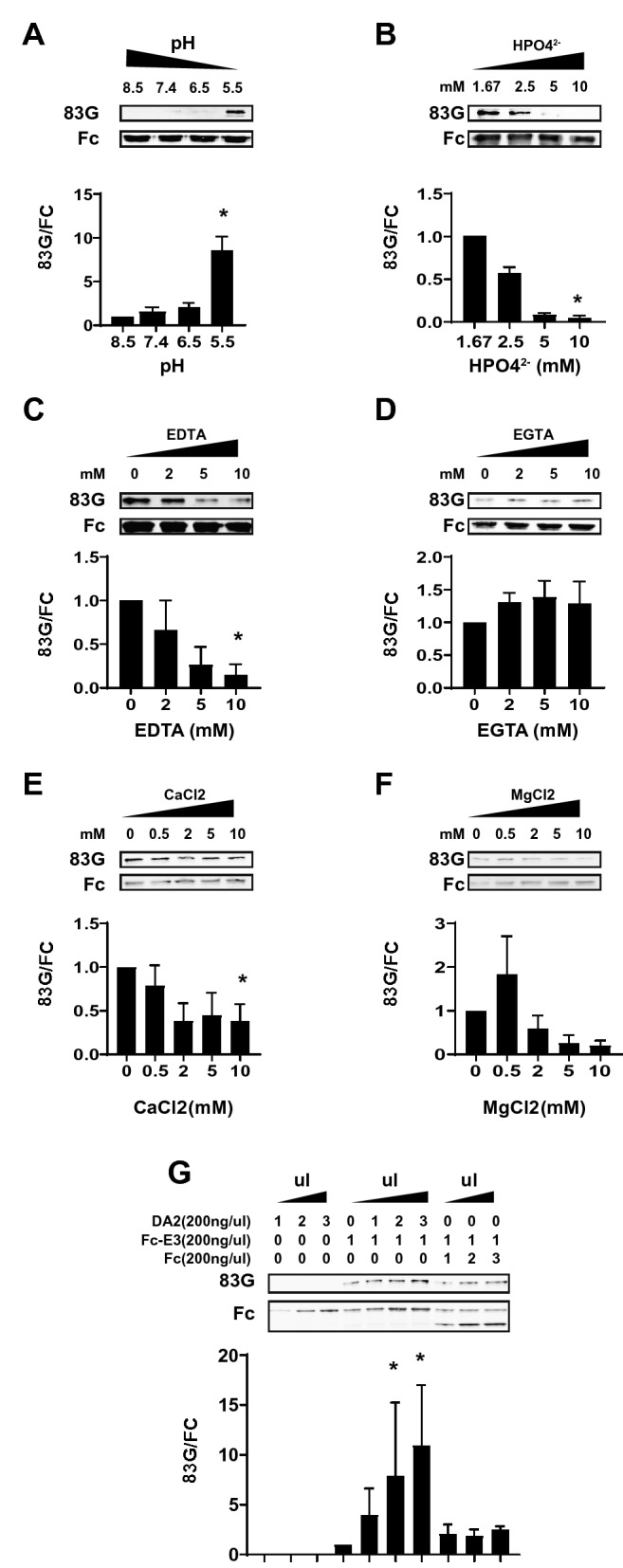
Effect of pH, ions, and protein concentration on NOTCH3 ectodomain cleavage at Asp121. Unless noted, all studies were performed with purified Fc-E3 protein that was incubated at 37 °C for 60 min to generate cleaved protein. Proteins were analyzed by Western blotting for Asp121 cleavage using 83G and total protein input was normalized to Fc that was probed on the same membrane. The amount of cut protein normalized to total input (Fc band) from three experiments was quantified in accompanying graphs. (**A**) Fc-E3 protein was incubated at indicated pH for 60 min at 37 °C. (**B**–**F**) Purified Fc-E3 protein was incubated at 37 °C at neutral pH in the presence of increasing concentrations of phosphate ion (**B**), EDTA (**C**), EGTA (**D**) calcium (**E**), and magnesium (**F**). (**G**) Effect of increased Fc-E3 protein on cleavage at Asp121. Increasing amounts of uncleavable mutant Fc-E3 (DA2 with Asp121Ala mutation; see Fig. 5B) was incubated with fixed amounts of cleavable Fc-E3, incubated as above, and the mixture was analyzed by Western blotting with 83G for cut protein and Fc for total protein. As a control, increasing amounts of Fc protein were added to Fc-E3, and after incubation as above, the mixture was analyzed in the same fashion. Quantification of cut proteins is shown. Statistical significance (*) p < 0.05 is shown.

We also investigated the effect of several experimental conditions on cleavage at Asp121. In prior experiments, cleavage at Asp80 was regulated by pH. To determine the effect of pH on cleavage at Asp121, we analyzed purified Fc-E3 after incubation in a range of conditions. Proteins treated with the lowest pH demonstrated the greatest degree of cleavage as assessed by Western blotting with 83G (Fig. 7A).

In the course of our investigation, we also discovered that the cleavage reaction was substantially inhibited in PBS. Based on this observation, we determined that phosphate ion (Fig. 7B) inhibited the cleavage at Asp121.

The role of additional biological ions was investigated in a series of similar studies that included incubations of Fc-E3 protein with divalent cation chelators. EDTA (Fig. 7C) decreased cleavage at Asp121, but the calcium-specific chelator EGTA did not (Fig. 7D). The addition of calcium (Fig. 7E) above physiological concentrations (2 mM or higher) resulted in a decrease in cleavage. But the addition of magnesium (Fig. 7F) did not significantly affect cleavage.

In the course of our studies, we recognized that cleavage of Fc-E3 protein at Asp121 was directly influenced by protein concentration. To determine whether Fc-E3 protein concentration specifically promoted cleavage, we purified a mutant form of Fc-E3 in which Pro122 was mutated to alanine to prevent cleavage. This uncleavable protein, when added to fixed concentrations of Fc-E3, resulted in increased cleavage of the latter (center lanes; Fig. 7G). The cleavage of Fc-E3 at Asp121 was not stimulated by supplementation of Fc protein (lanes on right; Fig. 7G).

## Discussion

CADASIL is the most common inherited cause of cerebral small vessel disease and vascular dementia^1,2^. The development of molecular insights into the mechanisms of disease offers promise to identify targeted preventative strategies that are currently limited to generic recommendations. In this study, we demonstrate that the NOTCH3 protein, which is mutated in CADASIL, undergoes cleavage at Asp121 in the media of cerebral vessels.

Small vessel disease of the brain is considered a chronic and progressive condition that can cause dementia and contribute to other age-related dementing disorders such as Alzheimer’s disease. Cleavage of proteins into pathological molecules occurs in a multitude of other chronic neurodegenerative conditions (e.g. Abeta, Tau, TDP-43 and alpha synuclein cleavage^8–13^). As such, cleavage of NOTCH3 in regions of greatest pathology supports potential mutual mechanisms at play in neurodegenerative processes.

The NOTCH3 protein has been the focus of many investigations in CADASIL. In prior work, a form of NOTCH3 accumulates that is antigenically similar to protein exposed to strong reductants^6^. In addition, prior work demonstrates redox stimulated cleavage at the Asp80, demonstrating that multiple post-translational transformations occur in disease^7^. The current work demonstrates that yet another cleavage event occurs, and, like cleavage at Asp80, cleavage at Asp121 is regulated by redox. The redox regulation of multiple transformations is consistent with the idea that cysteine disulfide status drives pathogenesis, a speculation raised by the stereotyped cysteine altering mutations found in CADASIL patients^4^. The sensitivity of the cleavage process to denaturation further supports that local protein conformation is critical for cleavage and implies that a specific biochemical environment of the labile Asp-Pro bond is required for cleavage. Like the cleavage after the first EGF-like repeat of NOTCH3, proteolysis at Asp121 likely occurs without proteases as the hydrolysis occurred in purified preparations and was insensitive to broad spectrum protease inhibitors (data not shown). Only a fraction of protein is cleavable at Asp121, as none of the reaction conditions (for example, Figs. 5 and 6A) were able to completely convert protein to a cleaved form, raising the possibility that NOTCH3 can exist in multiple conformational states.

Cleavage of NOTCH3 at Asp121 is enhanced in CADASIL patients, as assessed by 145H staining. These studies also emphasize that this novel post-translational alteration of NOTCH3 is in some ways different from other modifications (e.g. it is enriched in leptomeningeal arteries over penetrating vessels). However, cutting at Asp121 co-localizes in the same regions as cleavage products of NOTCH3 at Asp80 and disease relevant conformations of NOTCH3 (Fig. 4A–F). The Asp121 cleavage product also deposits in regions of the vessel that feature collagen IV and denatured collagen (Fig. 4G–J), which is consistent with a potential role of the cleavage product in executing downstream pathological events.

Using a model system, we show that cleavage at Asp121 can be influenced by specific factors. Phosphate appears to strongly block cleavage, which suggests that regulation of vascular ion composition could alter proteolysis. EDTA resulted in blockade of the proteolysis, though adding EGTA, calcium or magnesium did not increase NOTCH3 cleavage. EDTA, like phosphate, is polyanionic, suggesting that multiply charged small molecules, rather than metal chelation, may block cleavage at Asp121. In contrast, the finding that increased concentrations of NOTCH3 ectodomain, which multimerize^22,23^, increases cleavage indicates that factors which prevent protein–protein interactions of NOTCH3 could slow down cleavage. Given the relatively simple structure of chemicals thus far found to block cleavage at Asp121, selection of candidate small molecule inhibitors of cleavage may be feasible.

A limitation of this study is that it is possible that antibodies may recognize different targets in histological applications and Western blot, and therefore it could be argued that the tissue antigen in CADASIL bound by the antibodies may not necessarily be cut protein. This possibility is mitigated by the clearcut preference for all three histological antibodies to bind to terminal Asp121 residue-containing polypeptides in four different contexts (dot blots, Western blots of GFP fusions, cell staining, and in vitro protein cleavage reactions). Supportive evidence for cleavage of NOTCH3 in CADASIL will include identification of an appropriately sized protein on Western blots from CADASIL brain, a goal that has so far proven elusive.

Notwithstanding this limitation, these studies will stimulate further inquiry to answer unresolved questions that include: (1) Are there specific biochemical conditions in diseased vessels that favor cleavage at Asp-Pro sites? (2) Can other endogenous small polyanionic compounds be used to block Asp-Pro cleavage? (3) Are other proteins in the vessel wall also liable to Asp-Pro cleavage? (4) Prior work on the peptide generated by hydrolysis of the first EGF-like repeat suggests that NOTCH3 fragments adopt oligomeric conformations and are targeted by catecholamine-mediated complex formation. Do Asp-Pro cleavage products possess special reactivity or binding properties that actively promote pathophysiology?

In summary, we determine that CADASIL arteries feature the site-specific cleavage of NOTCH3 ectodomain at Asp121. This process highlights potential post-translational protein alterations in brain arteries as markers of disease and as potential modifiable targets. The reagents used to detect this process and the features of this cleavage event that we describe herein could be useful in future investigations of small vessel disease.

## Methods

### Antibodies

The 83G, 120B, and 145H rabbit monoclonal antibodies were generated for this study by standard procedures which are detailed elsewhere^24^. GenScript performed all animal experiments which were reviewed by the GenScript IACUC (animal protocol ANT19-005) and were performed in accordance with ARRIVE guidelines (a control group of non-peptide immunized animals was not included as this in not required for antibody generating procedures). All experiments were performed in accordance with relevant guidelines and regulations; specifically, methods of euthanasia were consistent with the recommendations of the Panel on Euthanasia of the American Veterinary Medical Association. A peptide antigen was synthesized corresponding to residues 105–121 (SCRCPRGFRGPDCSLPD) of human NOTCH3 protein. Rabbits were immunized followed by in vitro splenic cells fusion to create hybridomas (GenScript). ELISA performed using hybridoma supernatants identified candidate antigen-reactive clones with positive reactions over 1:2000 (threshold > twofold over negative media) considered sufficient for selection for further study. Antibodies employed in this study were derived from hybridoma cell supernatants that were not purified prior to use. Concentrations of antibody stocks were: 83G (151 µg/ml), 120B (93 µg/ml), and 145H (60 µg/ml). Unless noted, IHC was performed using 1:100 dilution for 145H, and WB and dot blots were performed using 1:1000 dilution of 83G and 120B.

Although all antibodies had similar specificity for the terminal Asp121 residue containing epitope, we used 145H for immunohistochemistry because it gave the lowest background staining in non-vascular parts of the brain that have not been shown to express appreciable amounts of NOTCH3 by others^5^. A comparative study is shown in Supplementary Fig. 2. In Western blot analyses, we interchangeably used 83G and 120B because they both recognized NOTCH3 sequences ending at Asp121 with higher affinity than 145H (Supplementary Fig. 7).

Antibodies UMI-D and UMI-F^7^ and 2079^6^ have been previously described for use in IHC. Antibodies were generated by transient transfection of cDNA expression clones encoding the heavy and light chains of the respective immunoglobulins into 293 cells grown in T75 flasks using PolyJet (as specified by SignaGen Laboratories). The day after transfection, cells were switched to OptiMEM (Invitrogen) and grown for another three days. The undiluted media was used for cell staining. M3F7 from the Developmental Studies Hybridoma Bank (University of Iowa; PMID 6682465 cat# M3F7, supernatant; used at 1 µg/ml) recognizes type IV collagen. The collagen binding peptide B-CHP (3Helix, cat# B-CHP, Biotin Conjugate; 50 µM) has been previously used to detect conformationally altered collagen proteins by IHC^21^.

### Tissue and cell staining

Formalin fixed frontal lobes sections were obtained from autopsies of CADASIL patients who had cysteine altering mutations in NOTCH3^6,20,25,26^. Control samples were obtained from the Alzheimer’s Disease Center at the University of Michigan and the Brain Bank of the National Institute for Developmental and Childhood Disorders at the University of Maryland^25^. A summary detailing patient characteristics is shown in Supplementary Table 1. CADASIL and control samples were stained at the same time in a random order. Five micron sections underwent chromogenic immunohistochemical (IHC) staining using rabbit antibodies after citrate-mediated antigen retrieval; sections were incubated in blocking solution (2% BSA in PBS) for 30 min then primary antibody was applied overnight in a humidified chamber at room temperature. Biotinylated secondary antibodies in blocking solution at 1:200 dilution were applied for 30 min followed by 15 min incubation of ABC solution prepared according to the manufacturer’s protocol (Vectastain Elite ABC kit, Vector Lab, cat# NC9293436). Finally, DAB incubation for the color reaction was applied for 1–5 min with the ImmPACT DAB HRP Substrate kit (Vector Lab, cat# NC9567138); all washing steps were done with running tap water, three to five times; after IHC, sections underwent hematoxylin counterstaining. Human tissue antigen integrity was confirmed by staining sections from each tissue sample with BRIC231 (anti-H; Santa Cruz, cat# sc-59467, 200 µg/ml Supplementary Fig. 8).

Immunocytochemistry was performed on 293 cells that were transfected with plasmids after fixation of cells with formalin. The same procedure was used as in IHC, except that all washes were done with PBS and that the cells were not counterstained with hematoxylin.

B-CHP staining was performed using the manufacturer’s recommended protocol (50 µM; 3Helix). The same procedure as above for IHC was used, except that there was no secondary antibody step. Briefly, the probe was heated prior to application on samples. All samples underwent heat-mediated antigen retrieval, a condition that denatures all collagens and results in B-CHP recognition of total collagen in the tissue.

To highlight elastic fibers of the arteries, Miller’s stain (EMS 26076-05) was used undiluted as recommended by the vendor on slides adjacent to immunostained sections.

Quantification of IHC was performed by two investigators blinded to sample and to each other’s scores. Scoring of staining with 145H of the leptomeningeal arteries of one frontal lobe section was performed as follows: 0 for no medial staining; 1 for clear staining in less than half of arteries; 2 for clear staining in over half of arteries. Scores from the two investigators were averaged, and individual scoring results are shown in Supplementary Table 1.

Quantitative analysis of 145H staining within individual leptomeningeal arteries was performed using the Fiji version of ImageJ (https://imagej.net/Fiji). Images were color deconvoluted to isolate DAB from hematoxylin staining using the "H DAB" parameter set. Arterial layer staining was obtained by measuring the gray value of each region of interest without setting a threshold (lower values of gray correspond to higher levels of signal). Two regions of interest analyzed included the media and the adventitia, which were quantified from the same file. In cases where the border between the media and adventitia could not be determined, the midpoint between the outer and inner wall of the vessel was considered the transition point. The ratio of medial to adventitial staining was calculated using the following formula: 1 − (medial gray value/advential gray value).

### DNA constructs

Synthetic or PCR-generated fragments of human *NOTCH3* cDNA were inserted into the C-terminus of the EGFP open reading frame by standard ligation procedures with pEGFP-C3 (Clontech); all constructs were sequenced to validate the presence of a continuous open reading frame which included fragments of *NOTCH3* of different length and/or point mutations. The Fc-E(x) series of constructs was generated in pSecTag (Invitrogen) and included an immunoglobulin gene signal sequence, mouse IgG Fc fused at its C-terminus to a sequence encoding the first (x) EGF-like repeats of human NOTCH3. For example, Fc-E1 encoded Fc fused to the first EGF-like repeat of human NOTCH3, ending in Pro81; the Fc-E2 construct encoded the first two EGF-like repeats of human NOTCH3, ending in Pro122; the Fc-E3 construct encoded the first three EGF-like repeats, ending in Glu161. We used PCR employing mutant oligonucleotide primers (and nested PCR if applicable) to generate point mutations in clones. In the case of the Fc-E3 clone, which encodes Fc and the first three EGF-like domains of human NOTCH3, in studies examining the effect of Pro122, we mutated multiple cysteine residues of EGF-like domain 1 (the first three, fifth and sixth cysteines) to serine in order to increase the sensitivity of the assay for cleavage.

### Recombinant protein preparation and analysis of proteolysis

Human HEK293 cells grown to at least 90% confluence were transiently transfected using PolyJet (SignaGen, cat# SL100688) using to the manufacturer's recommended protocol^24^. NOTCH3-encoding constructs were mixed with vectors that encode puromycin resistance, and the resulting pool of cells was selected for resistance by supplementing growth media with the antibiotic; this process generally resulted in dozens of cell colonies which were individually selected and independently propagated. The conditioned media of these cells (in OptiMEM; Invitrogen, cat# 31985070) was collected and subjected to protein A agarose chromatography and acidic elution followed by immediate neutralization with Tris and dialysis with two exchanges of PBS. Purity of the protein preparations was validated using silver staining.

For proteolysis studies, unless noted, 200 ng of purified proteins (at 200 ng/µl) were diluted in water and supplemented with chemicals specified in each experiment. In cases where no supplements were added, proteolytic assays were performed by incubated the diluted protein at 37 °C for one hour followed by analysis by Western blotting for cleavage using neo-epitope antibodies. The antibody signal was normalized to total Fc signal in each condition.

### Western and dot blotting

We have previously described these methods^6,24^. Proteins were separated on SDS polyacrylamide gels, transferred to nitrocellulose with an iBlot 2 instrument (Invitrogen, method P0 20 V for 1 min/23 V for 4 min/25 V for 2 min), blocked in TBST supplemented with 5% milk, and then incubated with primary antibodies in TBST overnight at 4 °C. Secondary antibody incubation was performed in TBST at room temperature for 30 min. Washing after primary and secondary antibody incubations were performed three times using TBST at room temperature. Secondary antibody used included: Donkey anti-mouse IRDye 680RD (Li-Cor #926-68072, 1:10,000 dilution, AB_10953628) and Goat anti-rabbit IRDye 800CW (Li-Cor #926-32211, 1:10,000 dilution, AB_2651127). Dot blots were performed by spotting peptides synthesized by GenScript on nitrocellulose, followed by detection as before^6^. Peptides (1 µg/µL) in 1 μL of water were spotted on membranes and allowed to dry at room temperature. Membranes were blocked in TBST supplemented with 5% milk, and then probed with primary and secondary antibodies as described above for Western blots. We used a Li-Cor Odyssey Imager for blot imaging with detection settings at 700 nm and 800 nm and used Li-Core Image Studio software for data capture and quantification. Full blots are available in Supplementary Figs. 9–12.

### Statistics

Mann–Whitney U tests were used to compare the differences between two experimental groups. Kruskal–Wallis tests followed by Dunn multiple comparison post hoc analysis were used to compare the differences among three or more experimental groups (Prism 8 analysis software). A probability value < 0.05 was regarded as statistically significant.

## Supplementary Information

Supplementary Information.
