# Supplementary material for: Hydrolysis of a second Asp-Pro site at the N-terminus of NOTCH3 in inherited vascular dementia
Source: Sci Rep. 2021 Aug 26;11:17246. doi: 10.1038/s41598-021-96679-9 (PMC8390697; doi:10.1038/s41598-021-96679-9)
Supplement: Supplementary file 1 — Supplementary Information. [file 41598_2021_96679_MOESM1_ESM.pdf]

Hydrolysis of a second Asp-Pro site at the N-terminus of NOTCH3 in inherited vascular dementia

Xiaojie Zhang[1,2], Soo Jung Lee[1,2], and Michael M. Wang[1-3] *

From the [1]Departments of Neurology, [2]Molecular and Integrative Physiology, University of Michigan, Ann Arbor, MI 48109
[3]Neurology Service, VA Ann Arbor Healthcare System, Department of Veterans Affairs, Ann Arbor, MI 48105

Running Title: NOTCH3 cleavage in vascular dementia

Address Correspondence to: Michael M. Wang, 7725 Medical Science Building II Box 5622, 1137 Catherine St., Ann Arbor, MI 48109-5622, Tel. 734-763-5453; Fax 734-936-8813; E-Mail: micwang@umich.edu

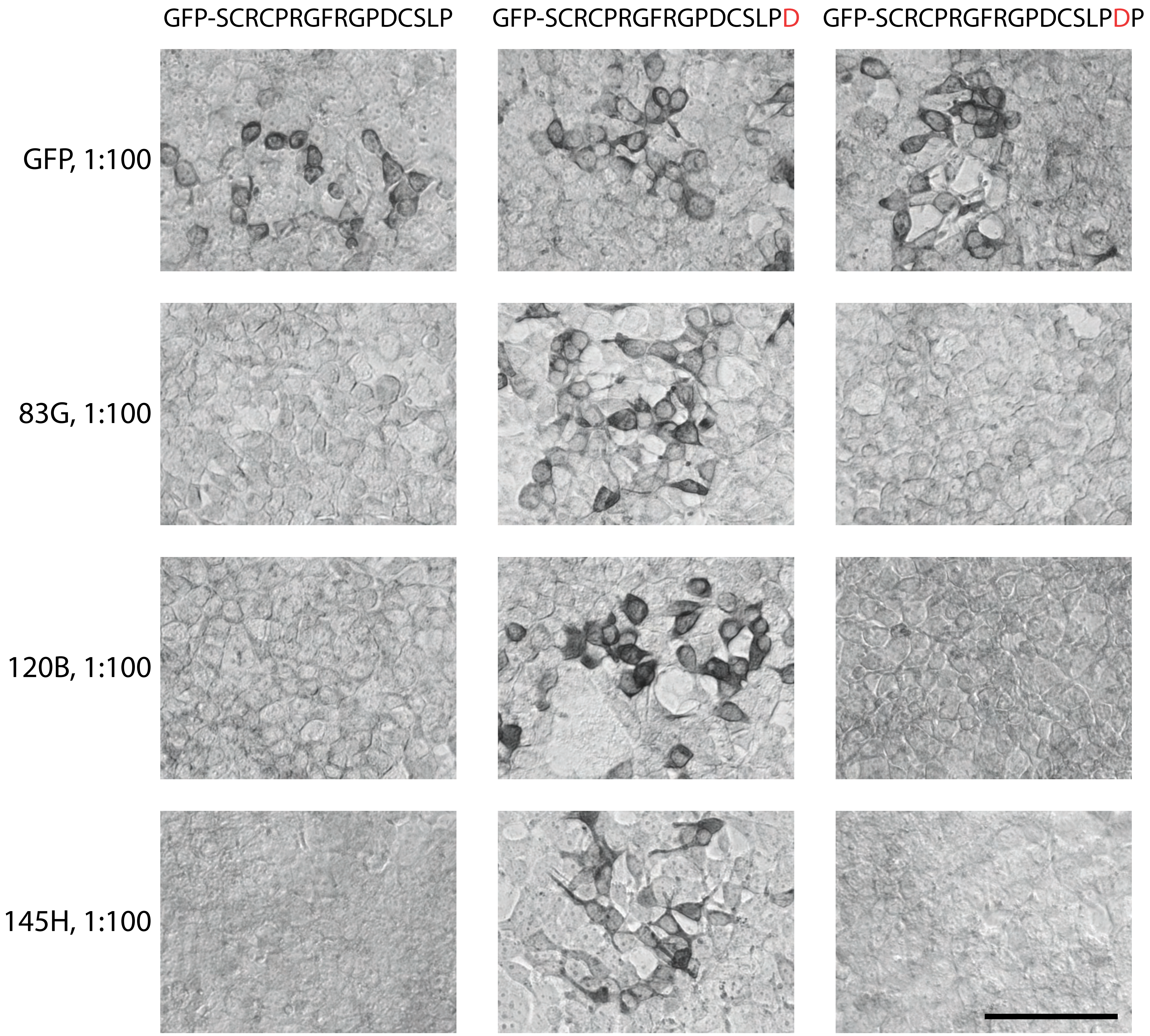

Supplemental Figure 1. Immunocytochemistry by 145H in 293 cells. 293 cells were transfected with GFP fusions ending in the EGF-like domain 2 sequences shown above each panel. Cells were then stained with either GFP antibodies, 83G, 120B or 145H. Top panels show GFP espression of all three fusion constructs was similar. Bottom panels show that staining with all three neo-epitope antibodies was selective for the epitope precisely ending in Asp121; none of the three antibodies recognize proteins with a deletion of Asp121 or with an extension beyond Asp121. The scale bar marks 100 microns which applies to all photos.

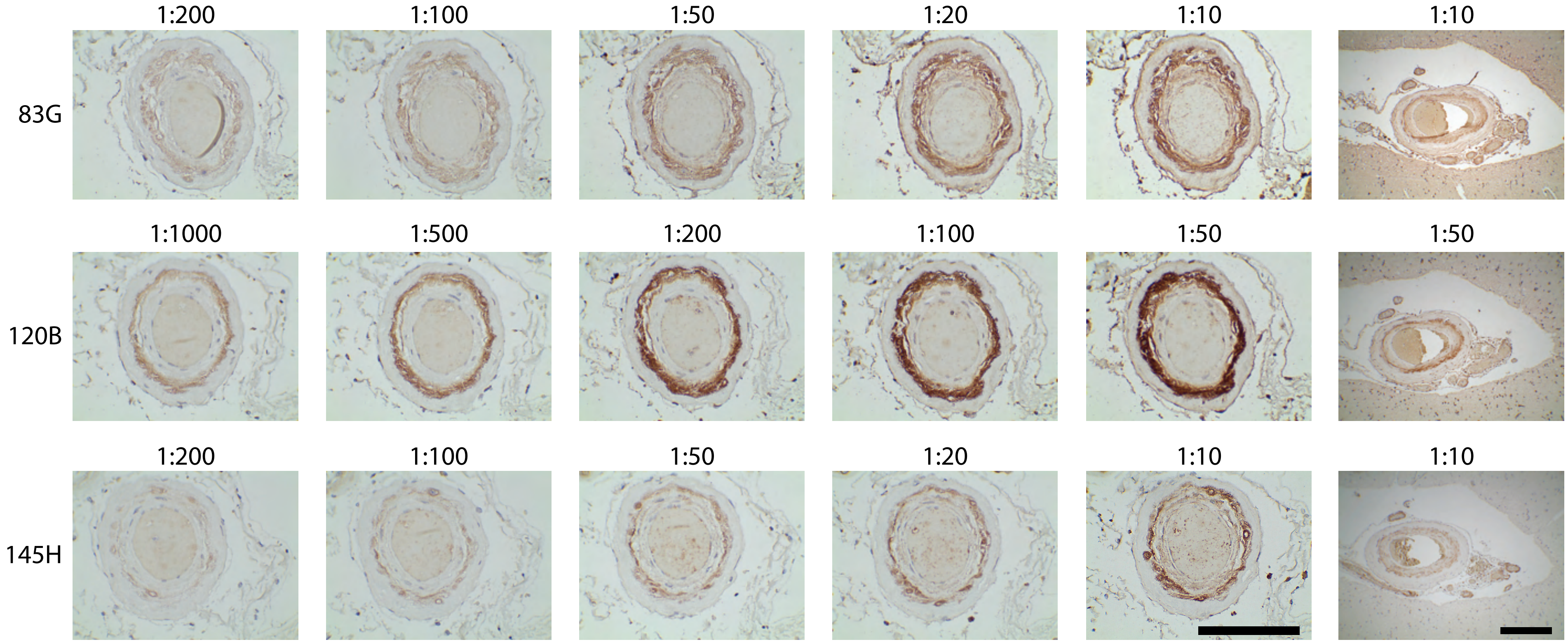

Supplemental Figure 2. Comparison of immunohistochemistry using serial dilutions of NOTCH3 neo-epitope monoclonal antibodies 83G, 120B, and 145H. Consecutive CADASIL sections were stained using 83G (stock 151 ug/ml), 120B (stock 93 ug/ml), and 145H (stock 60 ug/ml) with serial dilutions labeled above photographs. Leptomeningeal vessels showed gradients of increasing intensity of staining in the arterial media. Zoomed out views on the far right column show the background staining for each antibody. The large scale bar (100 microns) applies to all images except the those in the far right column. The small scale bar (100 microns) applies only to the images in the far right column.

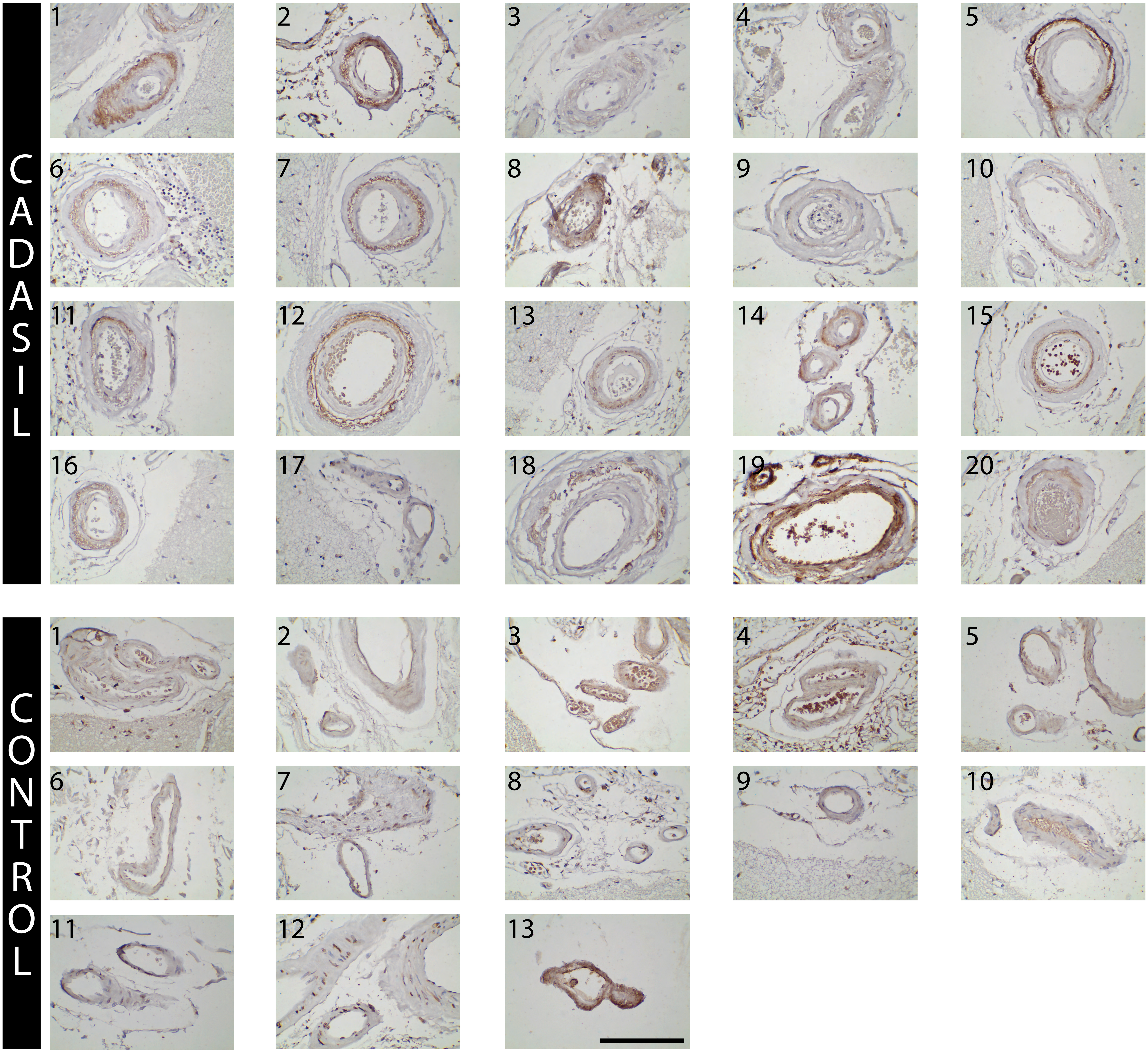

Supplemental Figure 3. Immunohistochemical staining of 20 CADASIL samples and 13 controls with antibody 145H. Paraffin sections were stained with antibody 145H at 0.6 ug/ml. Each panel focuses on leptomeningeal vessels which demonstrated the most consistent staining overall. Representative images from each section were selected to provide evidence of the range of staining observed between samples. In controls, there was no staining of vascular tissue above background. The scale bar marks 100 microns which applies to all images. For CADASIL and control vessels shown, the average ratio of medial to adventitial staining were 0.22 +/- 0.09 and 0.10 +/- 0.05 ($p < 0.001$), respectively.

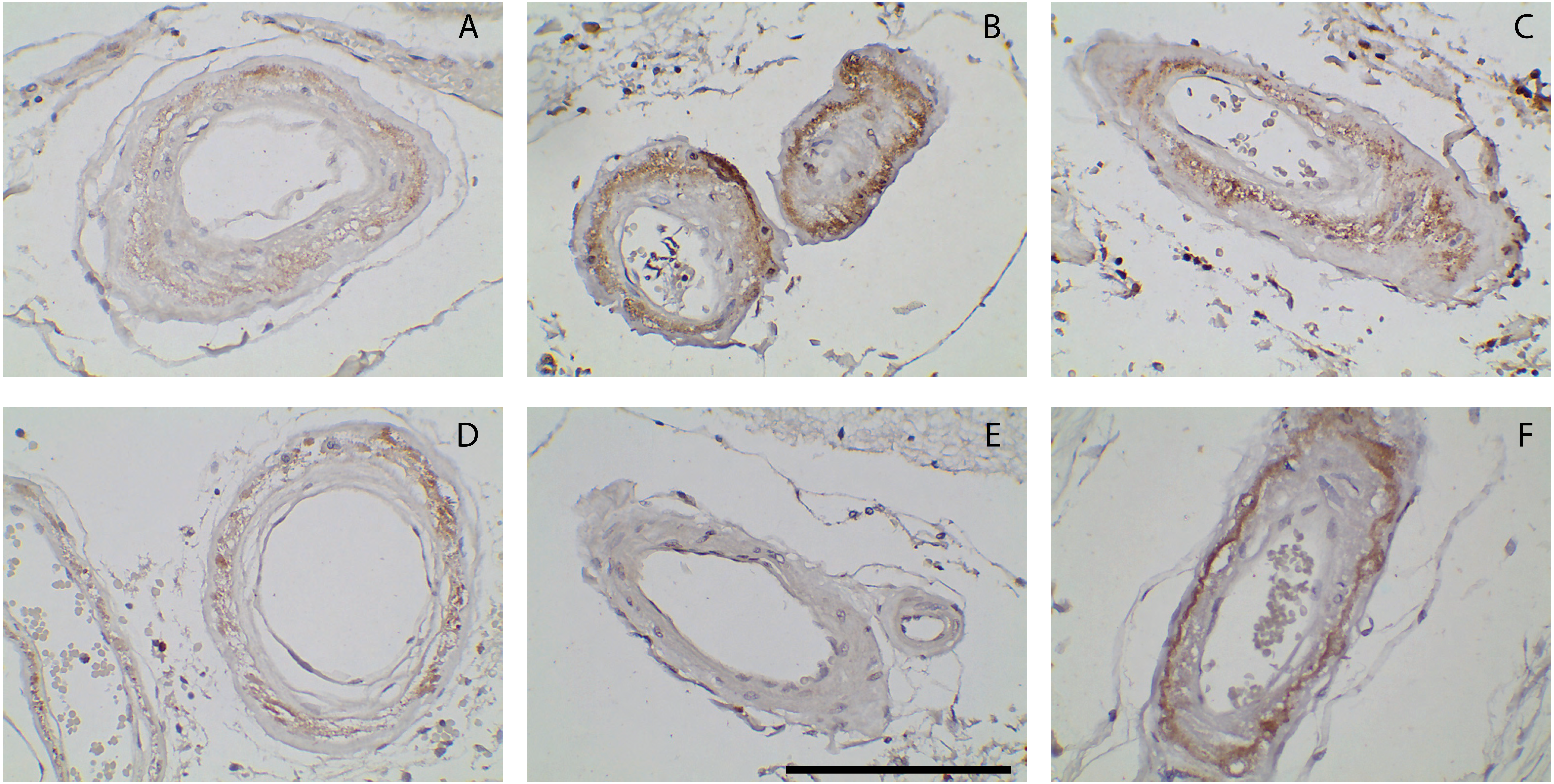

Supplemental Figure 4. Additional images of immunohistochemical staining of CADASIL samples with antibody 145H. Supplemental Figure 3 shows representative stained arteries from each CADASIL sample. In some samples, light staining intensity was the most common finding. Thus, stronger stained images were selected here to permit evaluation of 145H distribution patterns observed in lighter stained CADASIL samples. Staining using 145H for samples #3, 4, 9, 10, 17 and 18 from Supplemental Figure 3 are shown in panels A, B, C, D, E, and F, respectively. Sample #17 (E) is the only CADASIL sample out of 20 that did not stain with 145H.

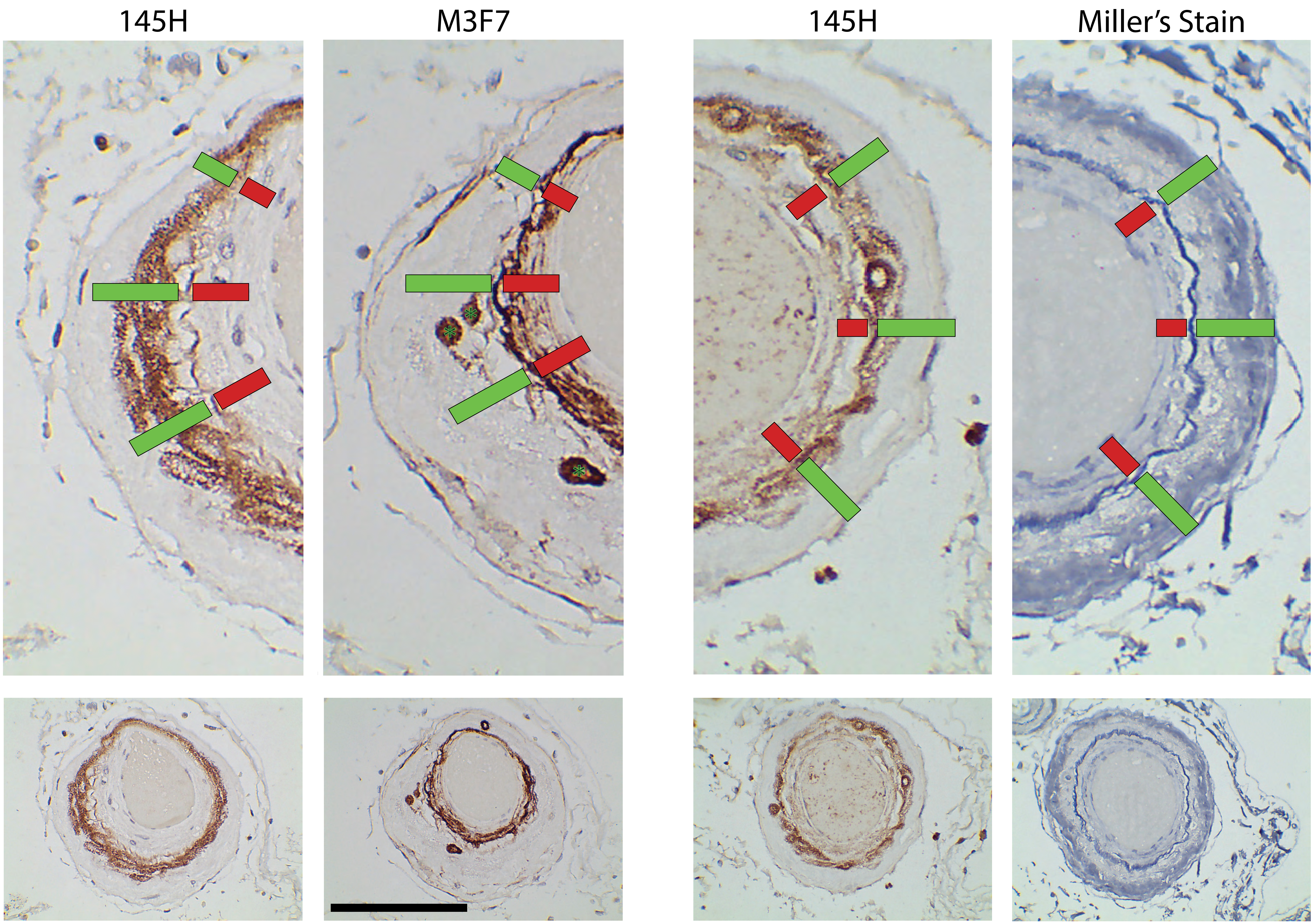

Supplemental Figure 5. Annotation of intima and media on a specific LM vessel of CADASIL in adjacent sections. The same images used in Fig 4A (145H for Asp121 cleavage, Fig 4G (M3F7 for type IV collagen), and Supplemental Fig 2 (145H at 1:10 dilution [6 ug/ml]) were expanded and annotated with a red bar for intima and a green bar for outer layer beyond the internal elastic lamina, which was evident throughout a majority of the circumference of the selected vessel. Miller's stain image clearly shows the elastic fibers in dark blue. The top images are derived from the smaller images place beneath each expanded image. Note that the 1:10 dilution of 145H was not the concentration used elsewhere in this study; it was chosen to compare the intimal to medial staining. The scale bar marks 100 microns for all images.

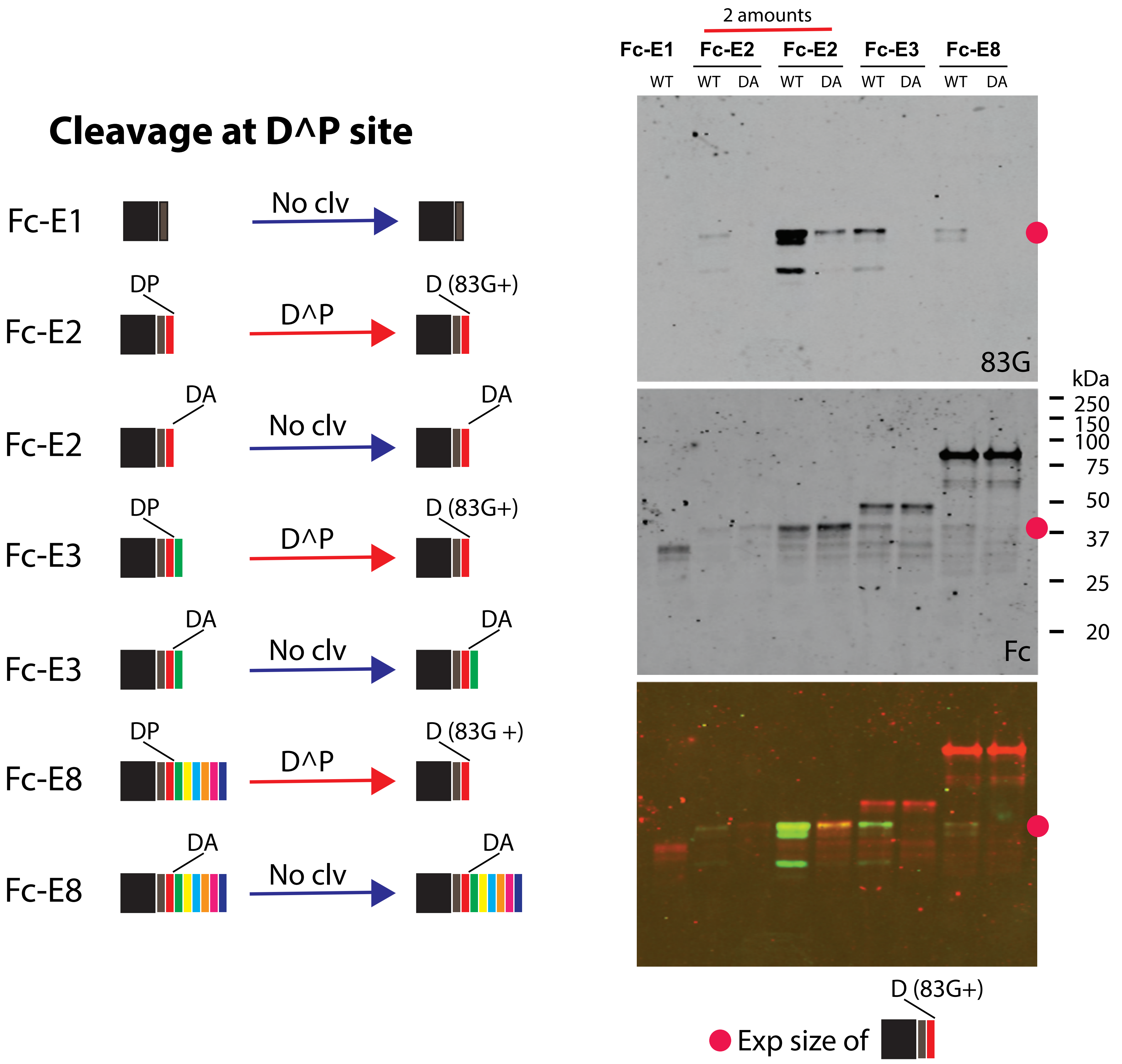

Supplemental Figure 6. A detailed schematic of proteins used in Fig 5B; their expected cutting efficiency is also shown, along with original blots from Fig 5B that has been annotated (red dot) to show the migration position of the cut protein detected by 83G (green bands).As noted, DP is the WT sequence between EGF-like 2 and 3 which is cut to release an epitope that is reactive with 83G. In the experiment shown, uncut proteins do not react with 83G. The DP to DA mutation reduces cutting at this site as shown in Fig 5 ("no clv"). D^P cleavage is never complete, resulting in residual uncut protein in every lane. 83G is more sensitive than anti-Fc (red bands) for cut protein, resulting in stronger 83G vs anti-Fc intensities of the cut products. The Fc-E2 cut (83G reactive) and uncut (anti-Fc reactive) products co-migrate because they are predicted to differ by only one amino acid.

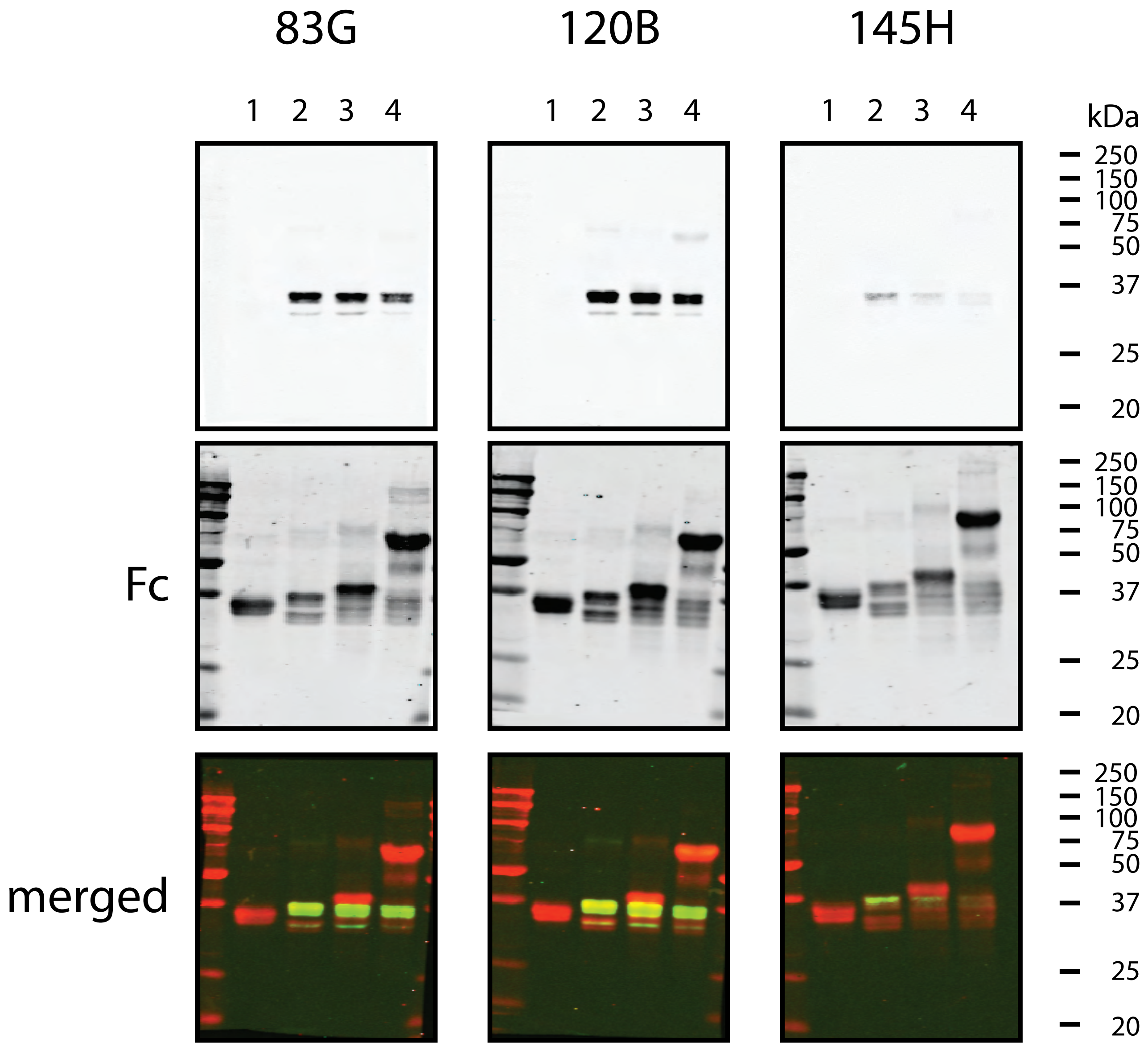

Supplemental Figure 7. Western blots using monoclonal antibodies 83G, 120B, and 145H to detect NOTCH3 cleavage at Asp121. Protein A-agarose purified recombinant proteins containing 1, 2, 3, or 8 EGF-like domains of NOTCH3 were separated on SDS-PAGE gels and analyzed by Western blotting. Lane 1: recombinant Fc-E1 proteins, Lane 2: recombinant Fc-E2 proteins, Lane 3: recombinant Fc-E3 proteins, Lane 4: recombinant Fc-E8 proteins. Schematic representations of the proteins used are shown in Fig 5B. In the top row, blots were probed with 83G, 120B and 145H antibodies. In lanes 2-4 (but not lane 1) all three antibodies recognized a fragment co-migrating with proteins that included only the first two EGF-like domains (Fc-E2 sized band). The blots were also probed for mouse Fc to show the migration patterns of the parent proteins, shown in the middle row. Colored figures in the lower panel show the merged images of protein recognized by monoclonal antibodies (green bands) and total Fc-containing proteins (red bands).

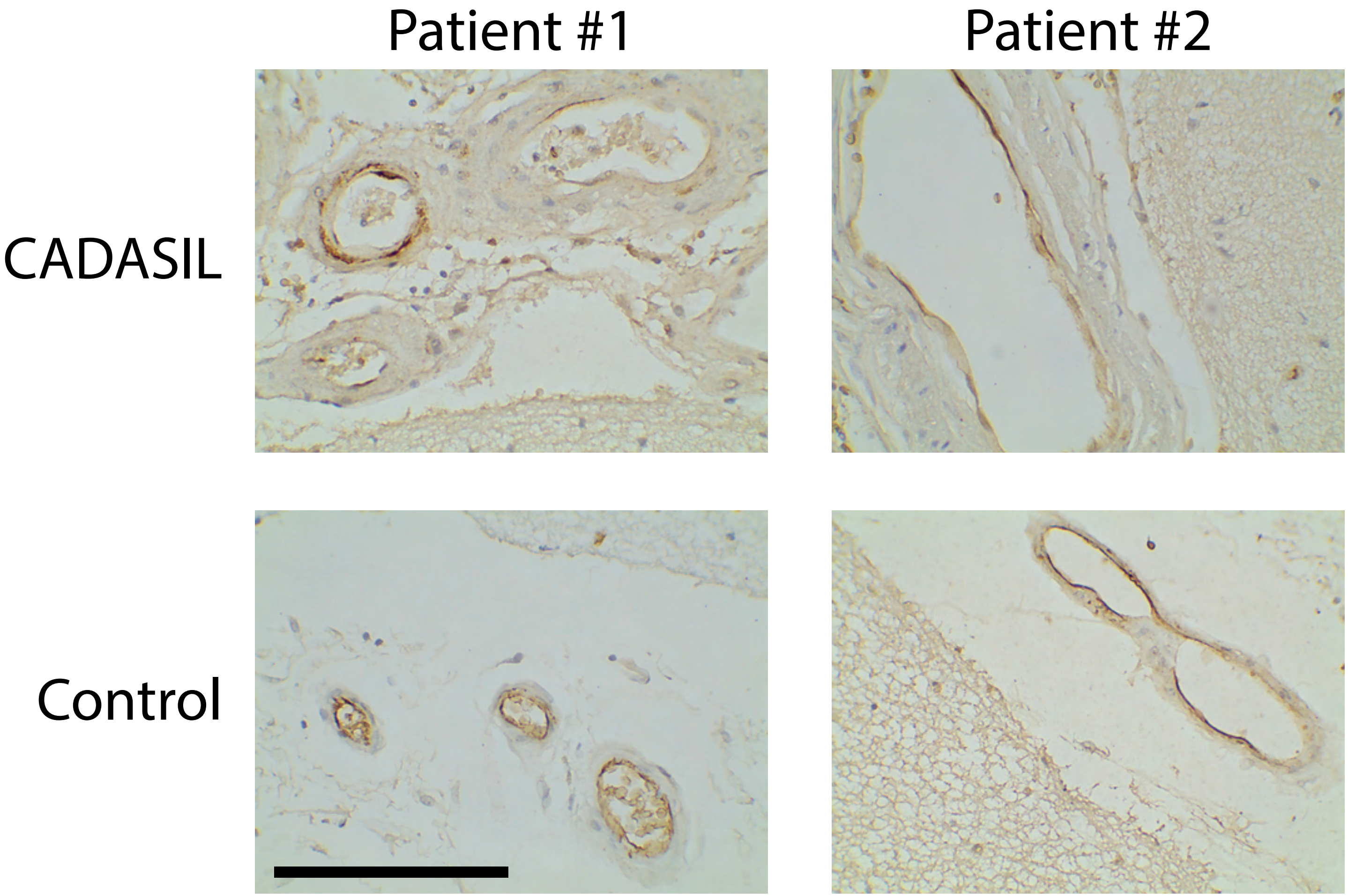

Supplemental Figure 8. Human tissue antigen integrity was confirmed by staining with BRIC231. Antigen integrity was assessed by staining tissues for control vascular antigens. Two representative leptomeningeal arteries from CADASIL and control tissues are shown which depict endothelial staining by anti-H antibody BRIC231 used at 2 ug/ml. The scale bar marks 100 microns which applies to all images.

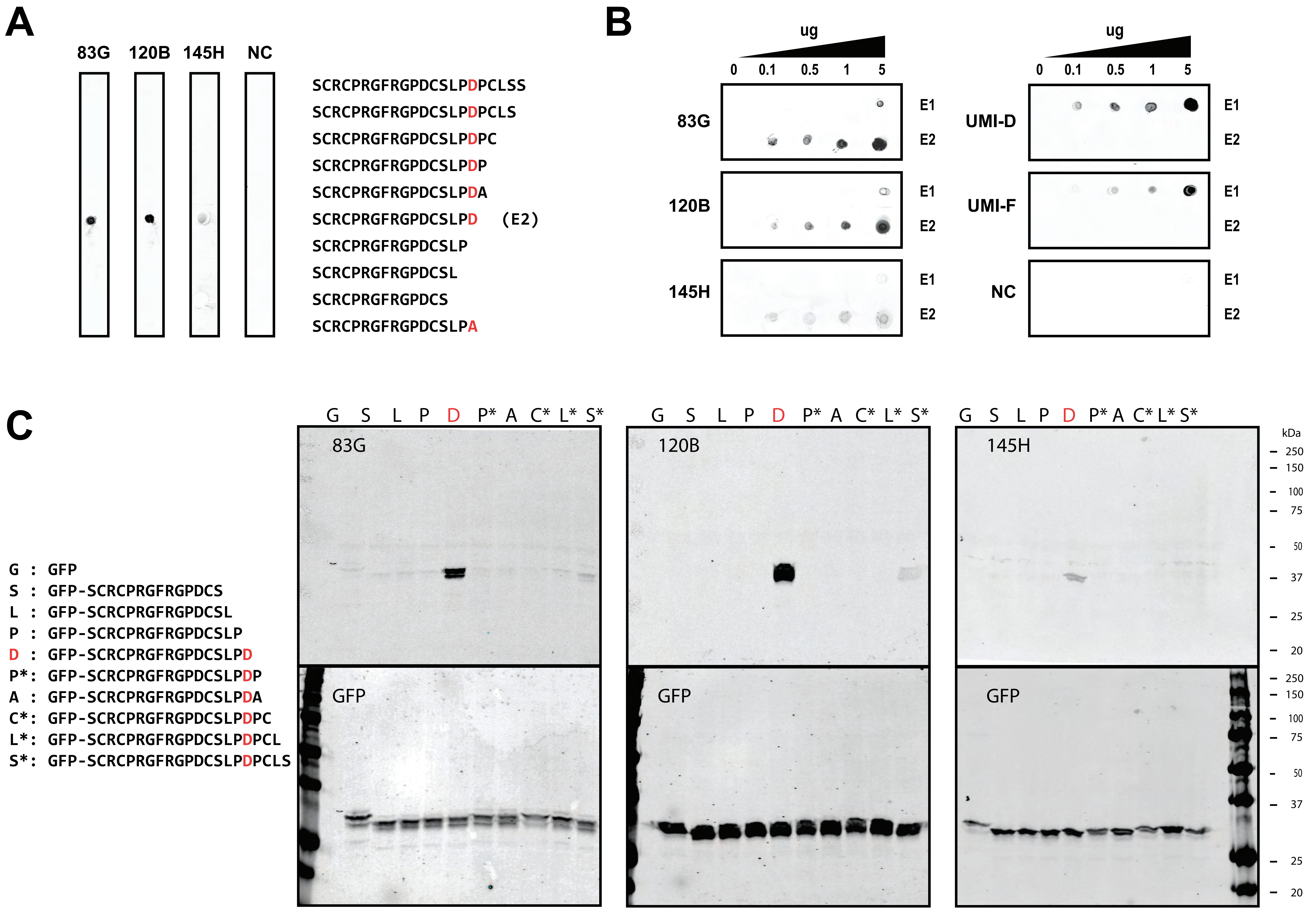

Supplemental Figure 9. Original blots for Fig 2 in the main text are provided. Please see figure legend in main text for details.

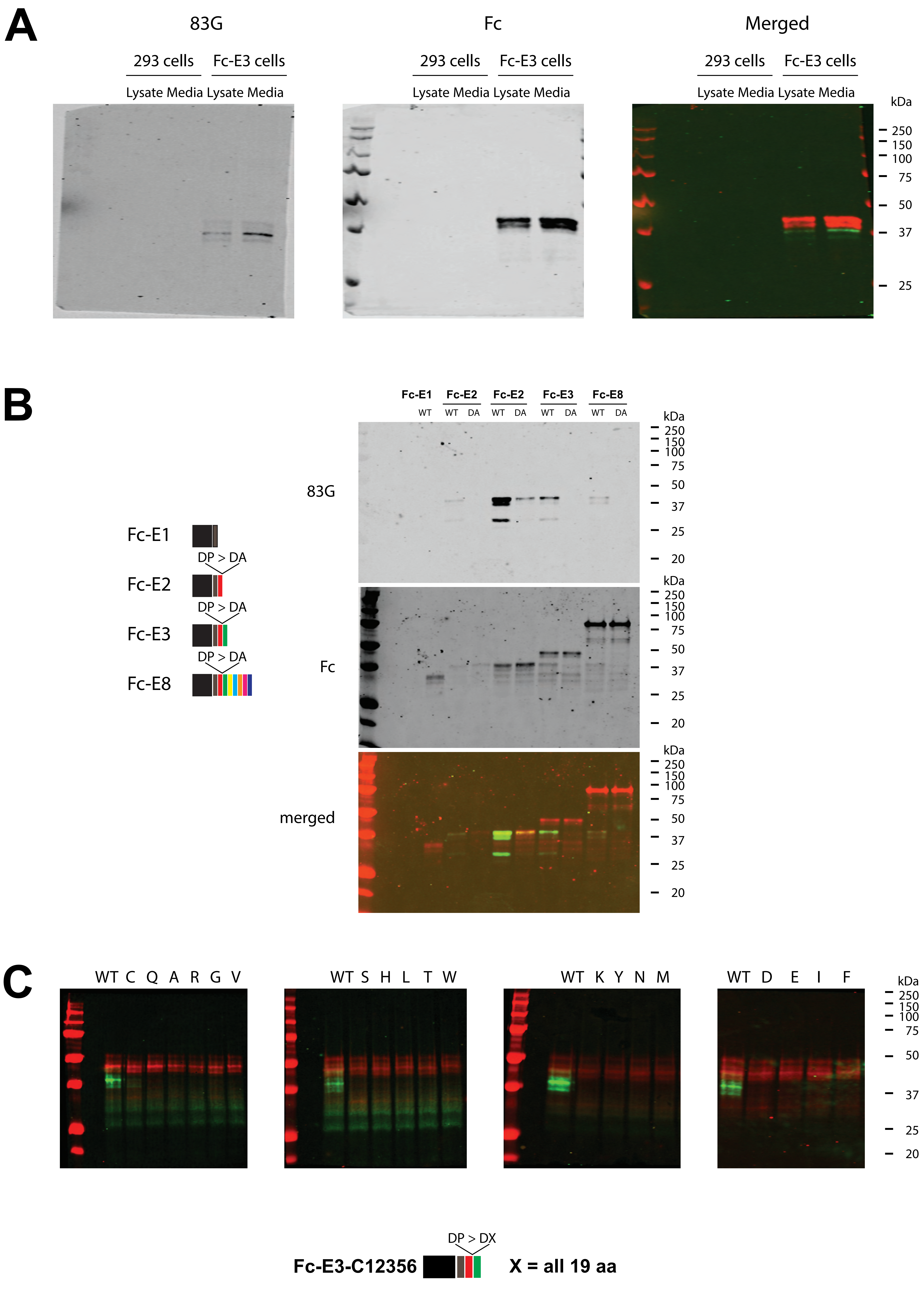

Supplemental Figure 10. Original blots for Fig 5 in the main text are provided. Please see figure legend in main text for details.

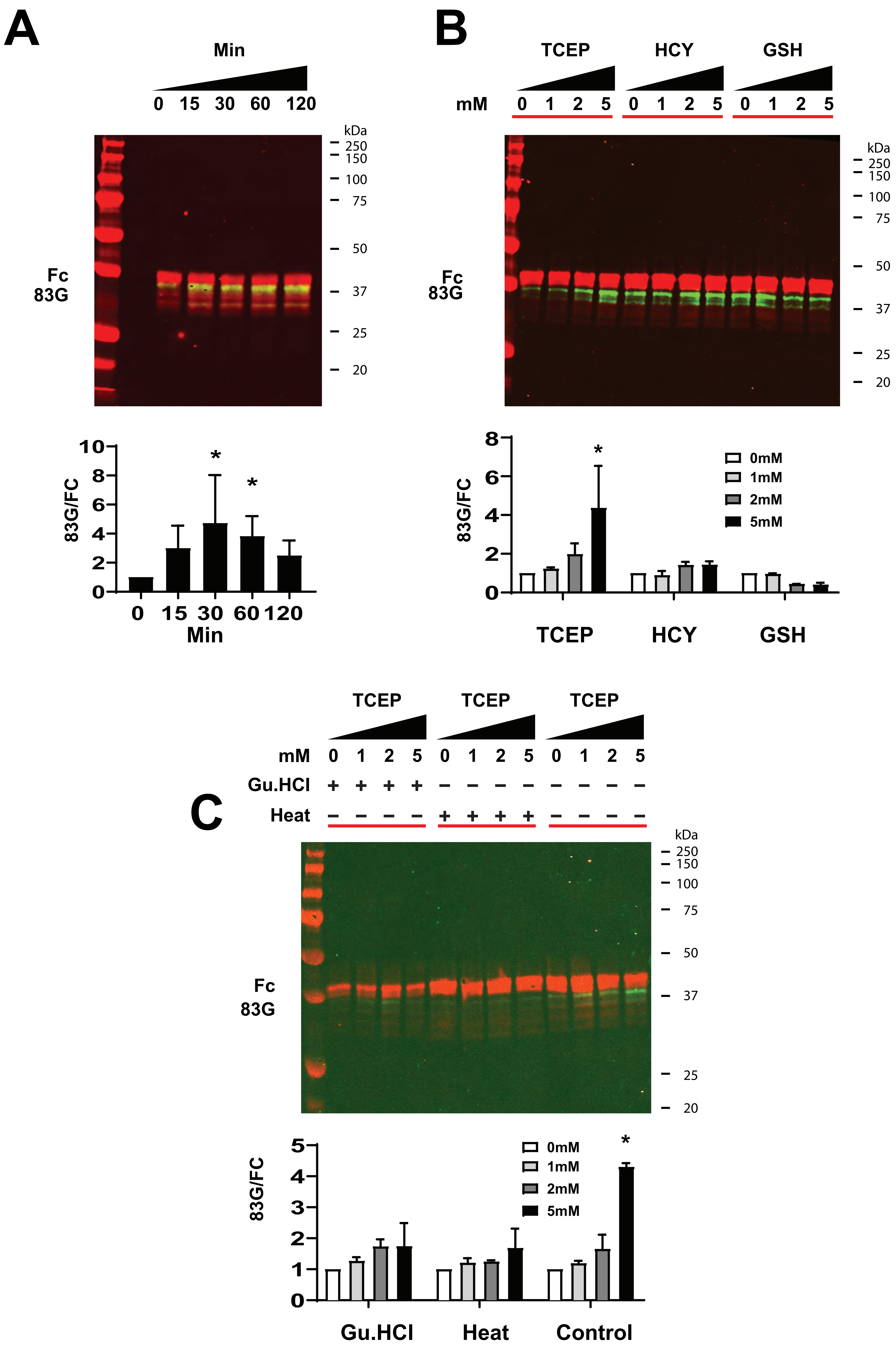

Supplemental Figure 11. Original blots for Fig 6 in the main text are provided. Please see figure legend in main text for details.

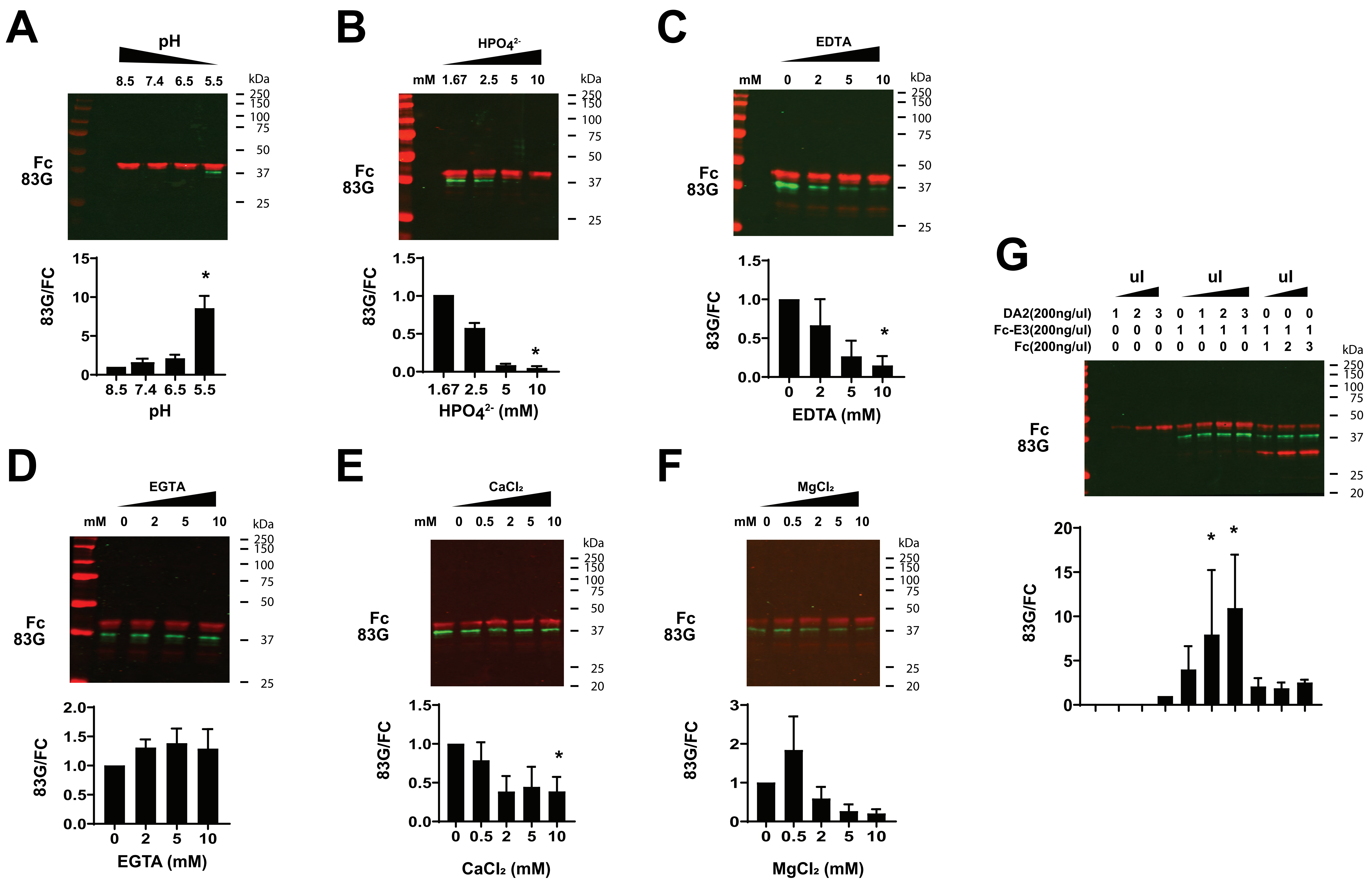

Supplemental Figure 12. Original blots for Fig 7 in the main text are provided. Please see figure legend in main text for details.

Supplemental Table 1. Characteristics of brain samples analyzed by immunohistochemistry. Twenty CADASIL samples were analyzed. All CADASIL samples were from post-mortem brain and originated from individuals with cysteine-altering genetic mutations in NOTCH3. Thirteen control samples were analyzed from brain banks listed in methods. Information from each sample is shown along with comments regarding general staining properties of the block (Notes). Representative staining using 145H of all patients are shown in Supplemental Figure 3. The scoring scale is described in the text; the scores from two independent analysts are shown. The final score was an average of both scores.

	Age (years)	Sex (f = female, m = male)	Post-Mortem Interval (hours)	Arterial staining scoring 1	Arterial staining scoring 2	Notes
CADASIL n = 20	50-59	M	34	2	2	
	50-59	M	9	2	2	
	40-49	M	45	2	2	
	50-59	M	42	1	2	
	60-69	F	36	2	2	High Bckgd
	60-69	M	59	2	2	
	60-69	M	15	2	2	
	60-69	F	5	1	2	
	40-49	F	35	1	2	
	40-49	M	55	1	2	
	50-59	M	21	2	2	
	70-79	M	60	2	2	
	50-59	M	23	1	2	
	70-79	M	19	1	2	
	70-79	F	16	1	2	
	70-79	M	9	1	2	
	50-59	M	42	1	2	
	60-69	M	32	1	1	
	40-49	F	38	1	1	
	60-69	M	21	0	0	

Non-CADASIL						
n = 13	0-9	M	12	0	1	
	80-89	F	9	1	1	
	70-79	M	10	0	0	
	50-59	M	17	0	1	
	40-49	M	6	1	1	
	10-19	F	5	0	0	
	80-89	F	5	0	0	
	0-9	F	20	0	0	High Bckgd
	80-89	M	23	0	0	
	60-69	M	22	0	0	
	50-59	M	18	1	0	
	60-69	M	15	1	0	
	60-69	F	30	2	2	High Bckgd
